# How possible is the development of an operational psychometric method to assess the presence of the 5-HTTLPR s allele? Equivocal preliminary findings

**DOI:** 10.1186/1744-859X-9-21

**Published:** 2010-05-07

**Authors:** Xenia Gonda, Konstantinos N Fountoulakis, Zoltan Rihmer, Andras Laszik, Hagop S Akiskal, Gyorgy Bagdy

**Affiliations:** 1 Department of Pharmacodynamics, Semmelweis University, Faculty of Medicine, Budapest, Hungary; 2 Department of Clinical and Theoretical Mental Health, Semmelweis University, Faculty of Medicine, Budapest, Hungary; 3 Third Department of Psychiatry, Aristotle University, University Hospital AHEPA, Thessaloniki, Greece; 4 Institute of Forensic Medicine, Semmelweis University, Faculty of Medicine, Budapest, Hungary; 5 Department of Psychiatry, University of California at San Diego, La Jolla, CA, USA

## Abstract

**Objective:**

The s allele of the 5-hydroxytryptamine transporter-linked promoter region (5-HTTLPR) polymorphism of the serotonin transporter gene has been found to be associated with neuroticism-related traits, affective temperaments and response to selective serotonin reuptake inhibitor (SSRI) treatment. The aim of the current study was to develop a psychometric tool that could at least partially substitute for laboratory testing and could predict the presence of the s allele.

**Methods:**

The study included 138 women of Caucasian origin, mean 32.20 ± 1.02 years old. All subjects completed the Hungarian standardised version of the Temperament Evaluation of the Memphis, Pisa, Paris, and San Diego Autoquestionnaire (TEMPS-A) instrument and were genotyped for 5-HTTLPR using PCR. The statistical analysis included the calculation of the Index of Discrimination (D), Discriminant Function Analysis, creation of scales on the basis of the above and then item analysis and calculation of sensitivity and specificity.

**Results:**

Four indices were eventually developed, but their psychometric properties were relatively poor and their joint application did not improve the outcome.

**Conclusions:**

We could not create a scale that predicts the 5-HTTLPR genotype with sufficient sensitivity and specificity, therefore we could not substitute a psychometric scale for laboratory genetic testing in predicting genotype, and also possibly affective disorder characterisation and treatment.

## Background

The s allele of the 5-hydroxytryptamine transporter-linked promoter region (5-HTTLPR) polymorphism of the serotonin transporter gene has been shown to be significantly associated with both unipolar, bipolar and subthreshold forms of affective disorder [1-8] and also the neuroticism trait [9-12], indicating a significant role of the polymorphism in the background of affective phenomena and pathology. In a previous paper we described that affective temperaments composing the depressive superfactor (that is, depressive, cyclothymic, anxious and irritable temperaments also show a significant association with the s allele) [13]. In a more recent paper, we attempted to compose a scale of those items of the Temperament Evaluation of the Memphis, Pisa, Paris, and San Diego Autoquestionnaire (TEMPS-A) scale measuring affective temperaments that differentiate most sensitively between subjects carrying and not carrying the s allele, and we managed to derive a scale consisting of nine items that was able to differentiate between the two groups at a good level of significance and also showed good internal consistency [14]. Since the s allele is associated not only with neuroticism and tendency to develop affective disorders in the face of adverse life events, but also with less favourable response to selective serotonin reuptake inhibitors (SSRIs) [15-19], we considered it of interest to develop a scale which could predict presence of the s allele to a high accuracy and thus less likely SSRI response. For this purpose a careful and meticulous psychometric approach is needed in delineating and validating the scale.

In the present paper we attempted to delineate and validate a scale based on the TEMPS-A questionnaire to predict the presence of the 5-HTTLPR s allele scale with a different and more rigorous approach.

## Methods

### Study participants

The study population included 138 psychiatrically healthy unrelated Hungarian women of Caucasian origin. All participants were aged between 18-64 years; the mean age of our subjects was 32.20 ± 1.02 years. All subjects were screened for neurological and psychiatric disorders using the standardised Hungarian version of the MINI International Neuropsychiatric Interview [20]. Subjects with any neurological and current or lifetime *Diagnostic and Statistical Manual of Mental Disorders, fourth edition *(DSM-IV) Axis I psychiatric disorders were excluded.

The study protocol was reviewed and approved by the Scientific and Research Ethics Committee of the Scientific Health Council of Hungary in charge of genetic experimentation concerning human subjects. All subjects gave written informed consent before participating in the study.

### Methodology

All subjects completed the Hungarian standardised version of the TEMPS-A questionnaire that measures affective temperaments on five scales, the depressive, cyclothymic, irritable, anxious and hyperthymic temperaments [14,21,22].

All subjects were genotyped for 5-HTTLPR by PCR. PCR amplification of 5HTTLPR was performed on genomic DNA extracted from buccal cells [23], and 5HTTLPR genotypes were identified as previously reported [24].

### Statistical analysis

All statistical analyses were carried out using Statistica 7.0 for Windows (Statsoft, Tulsa, OK, USA). In all cases we analysed our data according to the additive model (subjects with either of the three different genotypes: ss, sl, ll), according to the dominant model (subjects carrying the s allele and subjects not carrying the s allele), and according to the recessive model (subjects carrying the l allele vs subjects not carrying the l allele).

The first step included the calculation of the equivalent of the degree of difficulty [25] as a measure of an Index of Discrimination (D) in order to identify those items from the TEMPS-A scale that best discriminate groups. The D corresponds to the difference in the percentages in the responses given between two groups. The second step included the development of the scales with weighting the item responses; those with D above 15 were included in the scales with those with D above 20 weighted with a factor of 2, while those with D below 20 were weighted with a factor of 1.

Discriminant function analysis was also used in order to obtain two additional indices that could help in separating groups. All the items with D above 15 were included in this type of analysis.

Item analysis was performed, and the value of Cronbach's α for each scale was calculated. The sensitivity (Sn) and Specificity (Sp) were also calculated.

## Results

In all, 19 (13.76%) subjects carried the ss genotype, 50 (36.23%) the ll and 69 (50%) sl genotype. A total of 88 subjects (63.77%) carried the s allele while 50 subjects (36.23%) did not carry the s allele. The frequency of the s allele in our sample was 38.77% which parallels the results of earlier studies and is representative of the Caucasian population [24]. The distribution of genotypes in our study population followed the Hardy-Weinberg equilibrium (χ^2 ^= 0.38934, *P *= 0.8231).

The various genotype groups (ss, sl and ll) did not differ in age (*P *> 0.05) and they also did not differ concerning all the TEMPS-A subscales (Wilk's λ = 0.8833, F = 1.63, df = 10,262, *P *= 0.0980). However, post hoc comparisons indicated a significant difference in case of the Depressive, Anxious, Cyclothymic and Irritable subscales. When considering the presence or absence of the s allele (ss and sl combined vs the ll) then the difference concerning the TEMPS-A subscales was significant (Wilk's λ = 0.91, F = 2.40, df = 5,132, *P *= 0.0403) and concerned all individual subscales except the Hyperthymic (Depressive, Anxious, Cyclothymic, Irritable). The descriptive statistics are shown in Table 1.

**Table 1:** Descriptive statistics of the various study groups

		Dominant model: presence vs absence of s allele						Recessive model: presence vs absence of l allele								
				
		ss and sl (n = 88)			ll (n = 50)			sl and ll (n = 119)			ss (n = 19)			sl (n = 69)		
	
		Mean ± SD	Min	Max	Mean ± SD	Min	Max	Mean ± SD	Min	Max	Mean ± SD	Min	Max	Mean ± SD	Min	Max
Depressive		7.30 ± 3.18	2	16	5.98 ± 2.40	2	12	6.77 ± 3.05	2	16	7.11 ± 2.56	3	11	7.35 ± 3.35	2	16

Cyclothymic		6.23 ± 4.10	0	17	4.34 ± 3.01	0	11	5.62 ± 3.92	0	17	5.05 ± 3.34	0	10	6.55 ± 4.24	0	17

Hyperthymic		10.06 ± 3.67	1	20	10.72 ± 4.53	2	22	10.36 ± 4.09	1	22	9.89 ± 3.43	4	15	10.10 ± 3.75	1	20

Irritable		4.59 ± 3.53	0	15	3.30 ± 2.72	0	11	4.21 ± 3.41	0	15	3.58 ± 2.63	0	9	4.87 ± 3.71	0	15

Anxious		8.20 ± 5.25	0	19	5.94 ± 4.48	0	18	7.22 ± 5.10	0	19	8.42 ± 4.99	0	18	8.14 ± 5.35	0	19

ll scale		5.49 ± 2.85	0	11	3.24 ± 2.33	0	10	4.61 ± 2.89	0	11	5.05 ± 2.88	0	11	5.61 ± 2.85	0	11

ss scale		5.20 ± 2.46	1	11	4.12 ± 2.16	0	9	4.50 ± 2.25	0	11	6.74 ± 2.54	2	10	6.74 ± 2.54	2	10

The results from the calculation of D are shown in Tables 2 and 3. The resulting scale from the application of weighting on the selected items is shown in Table 4.

**Table 2:** Discrimination index (D) between the groups according to the dominant model (ss + sl vs ll) concerning the Temperament Evaluation of the Memphis, Pisa, Paris, and San Diego Autoquestionnaire (TEMPS-A) items

Dominant model (subjects carrying the s allele vs subjects not carrying the s allele)			
**TEMPS-A item**	**ss or sl (N = 88)**	**ll (N = 50)**	**D**

107A	51.14	28.00	23.14

17D	59.09	36.00	23.09

69I	45.45	26.00	19.45

7D	46.59	28.00	18.59

39C	36.36	20.00	16.36

94A	34.09	18.00	16.09

68I	31.82	16.00	15.82

29C	29.55	14.00	15.55

92A	53.41	38.00	15.41

27C	51.14	36.00	15.14

15D	38.64	24.00	14.64

99A	38.64	24.00	14.64

110A	52.27	38.00	14.27

100A	18.18	4.00	14.18

86A	34.09	20.00	14.09

89A	34.09	20.00	14.09

105A	50.00	36.00	14.00

52H	50.00	36.00	14.00

34C	31.82	18.00	13.82

35C	55.68	42.00	13.68

87A	29.55	16.00	13.55

4D	19.32	6.00	13.32

42C	19.32	6.00	13.32

64I	27.27	14.00	13.27

22C	43.18	30.00	13.18

73I	28.41	16.00	12.41

88A	34.09	22.00	12.09

24C	13.64	2.00	11.64

12D	29.55	18.00	11.55

23C	37.50	26.00	11.50

77I	27.27	16.00	11.27

90A	35.23	24.00	11.23

71I	20.45	10.00	10.45

33C	20.45	10.00	10.45

40C	36.36	26.00	10.36

66I	18.18	8.00	10.18

21D	26.14	16.00	10.14

The capital letter after the item number denotes the TEMPS-A subscale: A = anxious, D = depressive, C = cyclothymic or I = irritable.

**Table 3:** Discrimination index (D) between the groups according to the recessive model (ss vs sl + ll) concerning the Temperament Evaluation of the Memphis, Pisa, Paris, and San Diego Autoquestionnaire (TEMPS-A) items

Recessive model (subjects carrying the l allele vs subjects not carrying the l allele)			
**TEMPS-A item**	**ss (N = 19)**	**sl or ll (N = 119)**	**D**

98A	63.16	33.61	29.54

57H	63.16	42.02	21.14

55H	84.21	63.87	20.34

107A	57.89	40.34	17.56

71I	31.58	14.29	17.29

103A	63.16	47.06	16.10

89A	42.11	26.89	15.21

105A	57.89	42.86	15.04

91A	36.84	21.85	14.99

87A	36.84	22.69	14.15

110A	57.89	45.38	12.52

79I	21.05	9.24	11.81

33C	26.32	15.13	11.19

40C	42.11	31.09	11.01

16D	89.47	78.99	10.48

99A	42.11	31.93	10.17

15D	42.11	31.93	10.17

The capital letter after the item number denotes the TEMPS-A subscale: A = anxious, D = depressive, C = cyclothymic or I = irritable.

**Table 4:** Scale resulting from the application of weighting on the selected items of the TEMPS-A/5-hydroxytryptamine (5-HT) s allele subscale

Item	Criteria	Scoring
7D	I have always blamed myself for what others might consider no big deal	True = 1, False = 0

17D	I would rather work for someone else than be the boss	True = 1, False = 0

27C	I often blow up at people and then feel guilty about it	True = 1, False = 0

29C	My mood often changes for no reason	True = 1, False = 0

39C	I am the kind of person who can be sad and happy at the same time	True = 1, False = 0

55H	I love to be with a lot of people	True = 1, False = 0

57H	I am known to be generous, and spend a lot of money on other people	True = 1, False = 0

68I	I often feel on edge	True = 1, False = 0

69I	I often feel wound up	True = 1, False = 0

71I	I often get so mad that I will just trash everything	True = 1, False = 0

89A	Many people have told me not to worry so much	True = 1, False = 0

92A	I often feel jittery inside	True = 1, False = 0

94A	I often have an upset stomach	True = 1, False = 0

98A	When someone is late coming home, I fear they have had an accident	True = 1, False = 0

103A	I am, by nature, a very cautious person	True = 1, False = 0

105A	I easily get headaches when stressed	True = 1, False = 0

107A	I'm an insecure person	True = 1, False = 0

Index 1 (ss subscale): 2 × item 55 + 2 × item 57 + item 71 + item 89 + 2 × item 98 + item 103 + item 105 + item 107. Interpretation: score >6, highly likely for being an SS.

Index 2 (ll subscale): 2 × item 107 + 2 × item 17 + item 69 + item 7 + item 39 + item 94 + item 68 + item 29 + item 92 + item 27. Interpretation: index 2: score >3, highly unlikely for being an SS.

Index 3: If 1.19 × item 55 + 0.77 × item 57 + 0.94 × item 71 + 0.14 × item 89 + 1.14 × item 98 + 0.36 × item 103 - 0.02 × item 105 + 0.53 × item 107 - 4.38 <0 then it is highly unlikely to be an SS.

Index 4: If 0.09 × item 7 + 0.89 × item 17 + 0.34 × item 27 + 0.40 × item 29 + 0.79 × item 39 + 0.39 × item 68 + 0.26 × item 69 + 0.09 × item 92 + 0.26 × item 94 + 0.68 × item 107 - 0.91 <0 then it is highly likely to be either an SS or SL.

Cronbach's α was 0.48 for the ss and 0.66 for the ll scale. All items were more or less equal and omission of any of them did not alter the α value significantly.

The calculation of sensitivity (Sn) and specificity (Sp) at various cut-off levels for the two scales is shown in Table 5. The discriminant function analysis results are shown in Table 6. Both Sn and Sp as well as the discriminant function analysis results are poor and can not lead to the identification of cases. The combined use of these indices led to poor results as well since no case seemed to be classified by all the indices to the same allele category.

**Table 5:** Sensitivity and specificity of the two scales in discriminating between subjects carrying and not carrying the s allele

Score level	TP	TN	FP	FN	Sensitivity	Specificity
ss scale (subjects carrying the l allele vs subjects not carrying the l allele):						

4/5	15	62	57	4	78.95	52.10

5/6	13	81	38	6	68.42	68.07

5/6	13	81	38	6	68.42	68.07

6/7	11	97	22	8	57.89	81.51

ll scale (subjects carrying the s allele vs subjects not carrying the s allele):						

3/4	18	24	64	32	94.74	20.17

4/5	12	37	51	38	63.16	31.09

5/6	7	48	40	43	36.84	40.34

The ss scale discriminates between ss and combined sl and ll carriers (subjects carrying vs subjects not carrying the l allele; recessive model). The ll scale discriminates between ll and combined ss and sl carriers (subjects carrying the s allele vs subjects not carrying the s allele; dominant model).

FN = false negative; FP = false positive; TN = true negative; TP = true positive.

**Table 6:** Discriminant function analysis and development of the discriminating functions

ss scale				ll scale			
	Correct (%)	sl or ll	ss		Correct (%)	sl or ss	ll

ss	15.79	3	16	ll	56	28	22

sl or ll	99.16	1	118	ss or sl	85.23	13	75

Total	87.68	4	134	Total	74.64	41	97

The ss scale discriminates between ss and combined sl and ll carriers (recessive model). The ll scale discriminates between ll and combined ss and sl carriers (dominant model).

Function for ll scale: 0.09 × item 7 + 0.89 × item 17 + 0.34 × item 27 + 0.40 × item 29 + 0.79 × item 39 + 0.39 × item 68 + 0.26 × item 69 + 0.09 × item 92 + 0.26 × item 94 + 0.68 × item 107 - 0.91 <0 then it is either an ss or sl genotype.

Function for ss scale: 1.19 × item 55 + 0.77 × item 57 + 0.94 × item 71 + 0.14 × item 89 + 1.14 × item 98 + 0.36 × item 103 - 0.02 × item 105 + 0.53 × item 107 - 4.38 <0 then it is NOT an ss genotype.

All scales and indices correlated moderately but significantly with all TEMPS-A subscales (Table 7).

**Table 7:** Correlation matrix among the developed scales (ll subscale and ss subscale) and the TEMPS-A subscales

	ll subscale	ss subscale	Index 3	Index 4
TEMPS-A Depressive	0.64	0.38	0.27	0.57

TEMPS-A Cyclothymic	0.65	0.41	0.40	0.64

TEMPS-A Hyperthymic	-0.17	0.18	0.27	-0.17

TEMPS-A Irritable	0.48	0.34	0.39	0.46

TEMPS-A Anxious	0.71	0.66	0.53	0.58

All values are significant at *P *< 0.05.

## Discussion

In the present work we attempted to extract a scale from the TEMPS-A questionnaire that would predict the presence of the s allele of the 5-HTTLPR with satisfactory sensitivity and specificity. However, although several items discriminate between the different genotype groups to a high degree, no scale compiling these items showed high sensitivity and specificity with respect to the presence of the s allele. Even the combination of the scales that were derived cannot improve the poor classification outcome.

To understand the nature of psychometric disorders and to make more efficient treatment possible, we must not only view these disorders as complex entities in the context of their social, cultural, neurochemical and genetic determinants, but we should also be able to decompose psychiatric disorders into smaller and better characterisable components. The concept of endophenotypes was introduced to aim at identifying and characterising small, atomic phenomena that correspond to an accurately characterisable biochemical process or marker, such as a genetic polymorphism, and which is at the same time highly relevant in the manifestation of psychological phenomena or psychiatric disorders. There is an expanding effort to identify traits and temperaments related to the development of psychiatric illnesses and associate them with genetic factors. Studies have attempted to link psychological traits as measured by psychometric scales with a given polymorphism. Our approach in this case was different: based on an association we had already described between the 5-HTTLPR s allele and several affective temperaments measured by TEMPS-A [13,26], we aimed to construct a scale which would show a high ability to predict 5-HTTLPR genotype.

In a previous paper we attempted to solve the task of delineating a psychometric scale to predict presence of the s allele by selecting the items which differentiated between the different genotype groups using analysis of variance (ANOVA) and performing a subsequent item analysis [14]. In the current paper, however, we used a more rigorous statistical approach in selecting the items differentiating between the different genotype groups and calculated also sensitivity and specificity. As a result, we could not derive a scale that would predict the presence of the s allele with adequate accuracy.

The role of genetic factors in the background of personality, vulnerability and consequently psychiatric disorders has gained more recognition and wider acceptance in modern times. It is well accepted that the 5-HTTLPR s allele has a profound role in determining the emergence of neuroticism-related personality traits [9-12,27] and psychiatric disorders as well [1,2,4]. It has also been suggested and described in several studies that the presence of the s allele not only makes one more likely to possess personality traits which are associated with psychiatric diseases, especially anxiety and affective disorders, but it also makes a less favourable response to SSRI antidepressants more likely [15-17,28-31]. Understanding the underlying biological and personality factors profoundly shapes and reorganises how we view psychiatric disorders today and how they will be classified in the future. Also, these factors should be taken into consideration when selecting the appropriate treatment. Although genetic testing is an available and affordable procedure nowadays, it is not widely used due to several reasons including ethical factors. Moreover, the presence of a given polymorphic allele does not predict the manifestation of a given disorder, only indicates an increased risk. Similar is the case for drug response associated with genetic factors. Therefore a psychometric scale, which is short and easy to administer, and is able to predict presence of the genotype associated with certain personality factors, psychiatric disorders or response to drugs with a great specificity and sensitivity would be a useful tool not only in research but also in everyday psychiatric practice. In our study, however, we failed to develop such a scale, which indicates that as yet we have no accurate and useful psychometric tools that can substitute for biochemical laboratory testing. However, we report these scales in the current study in order to serve as a guide for future research and as they give a gross impression of the psychometric features associated with each genetic category.

In interpreting our results and drawing our conclusions, several limiting factors must be taken into consideration. First of all, our sample was relatively small; studies using larger samples would detect minor differences to a greater accuracy. Also, our sample consisted entirely of women. Further studies are needed to investigate the possibility of extracting a psychometric scale for predicting the s allele in men and in a mixed-gender general study population.

## Conclusions

Genetic polymorphisms influence not only the emergence of psychiatric diseases but also the pharmacotherapeutic response of these disorders to treatment. Although genetic polymorphisms only mildly contribute to such phenotypical alterations, they may be taken into account when selecting a pharmacological agent. A scale closely related to a given polymorphism may thus be a useful clinical tool, however, the development of such a scale needs further research.

## Competing interests

The authors declare that they have no competing interests.

## Authors' contributions

XG conceived the study, gathered and managed the data, performed the genetic analysis, participated in the statistical analysis and wrote the paper. KNF conceived the study, carried out the literature search and analysis and participated in writing the paper. ZR participated in analysing the data and writing the paper. AL participated in the genetic and statistical analysis. HSA participated in designing the study, analysing the data and writing the paper. GB participated in designing the study, analysing the data and writing the paper. All authors read and approved the final manuscript.
